# Effects of Co-Administration of Bone Marrow Stromal Cells and L-Carnitine on The Recovery of Damaged Ovaries by Performing Chemotherapy Model in Rat

**DOI:** 10.22074/ijfs.2019.5725

**Published:** 2019-07-14

**Authors:** Sam Zarbakhsh, Robabeh Safari, Hamid Reza Sameni, Behpour Yousefi, Manouchehr Safari, Nasrin Khanmohammadi, Parisa Hayat

**Affiliations:** 1 Nervous System Stem Cells Research Center, Semnan University of Medical Sciences, Semnan, Iran; 2 Cellular and Molecular Research Center, Iran University of Medical Sciences, Tehran, Iran

**Keywords:** Bone Marrow Stromal Cells, Carnitine, Chemotherapy, Ovary, Regeneration

## Abstract

**Background:**

L-carnitine (Lc) as a type of flavonoid antioxidants and bone marrow stromal cells (BMSCs) as a type of mesenchymal stem cells may recover damaged ovaries. It seems that Lc has favorable effects on differentiation, increasing lifespan and decreasing apoptosis in BMSCs. The aim of this study was to investigate effects of co-ad- ministration of BMSC+Lc on damaged ovaries after creating a chemotherapy model with cyclophosphamide in rats.

**Materials and Methods:**

In this experimental study, cyclophosphamide was intraperitoneally (IP) injected to forty female wistar rats for 14 days, in terms of chemotherapy-induced ovarian destruction. The rats were then randomly divided into four groups: control, Lc, BMSCs and co-administration of BMSC+Lc. Injection of BMSCs into bilateral ovaries and intraperitoneal injection of Lc were performed individually and together. Four weeks later, levels of se- rum estradiol (E2) and follicle-stimulating hormone (FSH) using enzyme-linked immunosorbent assay (ELISA) kit, number of ovarian follicles at different stages using hematoxylin and eosin (H&E) staining and expression of ovarian Bcl-2 and Bax proteins using western blot were assessed.

**Results:**

Co-administration of BMSC+Lc increased E2 and decreased FSH levels compared to the control group (P<0.001). The number of follicles was higher in the co-administrated group compared to the control group (P<0.001). Co-administration of BMSC+Lc increased Bcl-2 protein level, decreased Bax protein level and increased Bcl-2/Bax ratio (P<0.001).

**Conclusion:**

The effect of co-administration of BMSC+Lc is probably more effective than the effect of their separate administration on the recovery of damaged ovaries by chemotherapy.

## Introduction

Despite the great benefits of chemotherapy in treating cancer patients, it has some side effects on ovaries (1). Cytotoxic effects of chemotherapy damage the granulosa cells (GCs), so that folliculogenesis disruption may occur (2). Unfortunately, this issue is disappointing for girls and young women who receive chemotherapy. Cyclophosphamide is one of the most administrated chemotherapy drugs which directly affects ovaries (3). There are several methods to treat ovarian damage, including hormone therapy, freezing ovaries, stem cell therapy and applying antioxidants (4). Hormone therapy is not suitable for cancer patients, because it may increase the probability of the cancer recurrence (5). As disadvantages of ovarian cryopreservation, it requires surgical procedures for tissue harvesting and transferring, while probability of returning its function is low (6). Recently, it has been observed that transplantation of bone marrow stromal cells (BMSCs), a type of mesenchymal stem cells, may treat ovarian damage after chemotherapy (7, 8). BMSCs can produce some growth factors, differentiate into other cell lines and replace damaged cells (9, 10). On the other hand, it has been shown that some antioxidants such as L-carnitine (Lc) have beneficial effects on damaged ovaries (11). Lc is a flavonoid antioxidant that plays an essential role in fatty acid metabolism and is present in human serum and tissues (12, 13). However, the effect of Lc has not been assessed on damaged ovaries by chemotherapy.

Several reports have shown that Lc has favorable effects on mesenchymal stem cells, including suppression of apoptosis in BMSCs (14), modulating differentiation of adult mesenchymal stem cells (15) and improvement of the aged adipose tissue-derived human mesenchymal stem cells lifespan (16).

Although the effects of individual BMSCs and Lc on the repair of damaged ovaries have been investigated, there is no report yet concerning the effect of simultaneous administration of them on the recovery of damaged ovaries. So, in this study, due to the beneficial effects of Lc on BMSCs, we evaluated for the first time the effect of co-administration of BMSC+Lc on ovarian function and structure after creating a chemotherapy model with cyclophosphamide in rats.

## Materials and Methods

### Animals

In this experimental study, forty female wistar rats (180- 200 g) were used. They had free access to food and water under controlled temperature (25 ± 2.C). Vaginal smear was daily obtained and only those showing at least two consecutive normal vaginal estrus cycles were used in the experiments. All procedures were approved by the Research Council of Semnan University of Medical Sciences (Semnan, Iran). The Ethical Code is IR.SEMUMS.REC.

### Bone marrow stromal cell culture and characterization

After sacrificing an adult rat, femurs and tibias were dissected out. Bone marrow was ejected with 10 ml of Dulbecco’s Modified Eagle Medium (DMEM) and cultured in DMEM containing 10% fetal bovine serum (FBS) and 1% penicillin/streptomycin (all from Gibco, Germany), incubated at 37.C, 95% humidity and 5% CO_2_. After 48 hours, non-adherent cells were removed by replacing the medium. The cells were sub-cultured four times (17, 18).

To analyze expression of the stem cell surface markers, at least 100,000 cells were incubated with fluorescence- labeled monoclonal antibodies against CD29, CD34, CD44, CD45 and CD90 (Sigma, China). Following a 10 minutes wash in phosphate-buffered saline (PBS, Sigma, USA), the labeled cells were analyzed using a Becton Dikinson FACS Calibur Flow Cytometer (BD, USA) (7).

### Creating the chemotherapy model

To destroy the ovaries, a model of chemotherapy was created. Cyclophosphamide (Sigma, China) diluted in normal saline was intraperitoneally (IP) injected at 50 mg/ kg at the first day, followed by 13 days injection of 8 mg/ kg daily cyclophosphamide (19).

### The injection procedure in the groups

After creating the chemotherapy model, the rats were randomly divided into four groups (n=10 in each group): i. Control group, 25 .l of culture medium was directly injected into the bilateral ovaries, ii. BMSC group, 2×10^6^ BMSCs suspended in 25 .l culture medium were directly injected into the bilateral ovaries (20), iii. Lc group, 200 mg/kg of Lc was injected IP, one day before beginning chemotherapy, until 7 days after chemotherapy (11), and iv. BMSC+Lc co-administrated group, combined BMSCs and Lc was injected.

### Bone marrow stromal cell tracking in the ovaries

To track the transplanted BMSCs after four weeks in the ovaries, the cells were labeled with DiI (1,1’-dioctadecyl- 3,3,3’,3’-tetramethyl indocarbocyanine perchlorate) (Sigma, China). Briefly, BMSCs were suspended in DMEM and 5 .l/ml DiI was added. After incubation for 20 minutes, the cells were centrifuged and washed with PBS, and then suspended again for transplantation. Four weeks after transplantation, prepared paraffin sections and the labeled cells were detected by fluorescence microscope (Motic, Spain) (21).

### Hormonal evaluation

Four weeks after the end of chemotherapy, serum estradiol (E2) and follicle-stimulating hormone (FSH) levels of these groups were measured by enzyme-linked immunosorbent assay (ELISA) kits (East Bio-Pharm, China) for rat, according to the manufacturer’s instruction (22).

### Histological evaluation of the ovaries

Four weeks after the end of chemotherapy, the ovaries were collected and fixed in 4% paraformaldehyde, dehydrated, paraffin-embedded and serially sectioned at 5 .m thickness. Five representative sections from each ovary were randomly chosen and routine hematoxylin and eosin (H&E) staining was performed for histological examination with light microscopy. the number of primordial, primary, secondary and antral follicles were measured (1).

### Western blot assays

Five ovaries in each group were lysed using RIPA buffer (Cell Signaling Technology, Netherlands) supplemented with protease inhibitor (Roche, Switzerland) on ice for 30 minutes. Then, the mixture was centrifuged at 13000 rpm for 20 minutes at 4°C. Equal value of proteins (80 .g) were loaded on sodium dodecyl sulfate (SDS, Sigma, Japan) polyacrylamide gel (Merck, Germany) and separated in a size manner by electrophoresis. The proteins were transferred to nitrocellulose membranes (Amersham Biosciences, USA). The membranes were blocked with 5% skim milk in tris buffered saline (TBS, pH=7.4). The membranes were incubated with primary antibodies for Bcl-2 (1:1000), Bax (1:1000) and .-Actin (1:1000, Abcam, USA) overnight at 4°C. After washing, the membranes were incubated with goat anti-rabbit secondary antibody conjugated with horseradish peroxidase (HRP). All antibodies were diluted according to manufacturer’s instructions. Immunoreactive bands were visualized using an enhanced chemiluminescence detection system (Amersham Biosciences, USA). X-ray films were scanned, and then the relative protein levels were semi-quantified by densitometric analysis using image j software. .-actin was tested as the internal control (23).

### Statistical analyses

After verifying the normality of variance assumptions, data were analyzed by one-way analysis of variance (ANOVA) followed by the Tukey Test. Obtained data are presented as the mean ± SE, and a level of P<0.05 was considered statistically significant.

## Results

### Cultivation and characterization of bone marrow stromal cells

**Fig 1:**
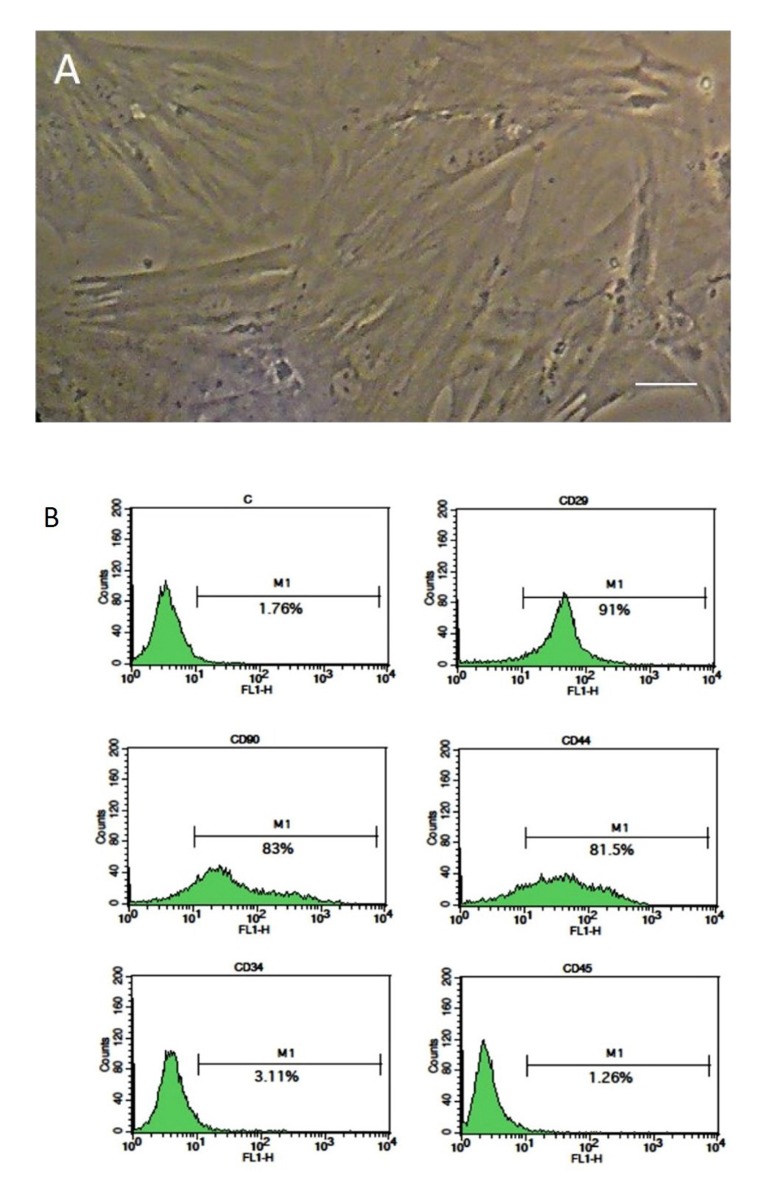
Isolation and identification of bone marrow stromal cells (BMSCs). **A.** Cultured BMSCs at passages 4 and **B.** The results of flow cytometry show that BMSCs are positive for CD29, CD44 and CD90, while it is negative for CD34 and CD45 (scale bar: 50 .m).

BMSCs were cultured in the T25 flasks. After a few days, the cells appeared to be spindle-shaped. By repeating passages, the cells became morphologically homogeneous. Most of the cells expressed the mesenchymal stromal cell markers (CD29, CD44 and CD90) and did not express the hematopoietic cell markers: CD34 and CD45 (Fig .1).

### Bone marrow stromal cell tracking in the ovaries

**Fig 2:**
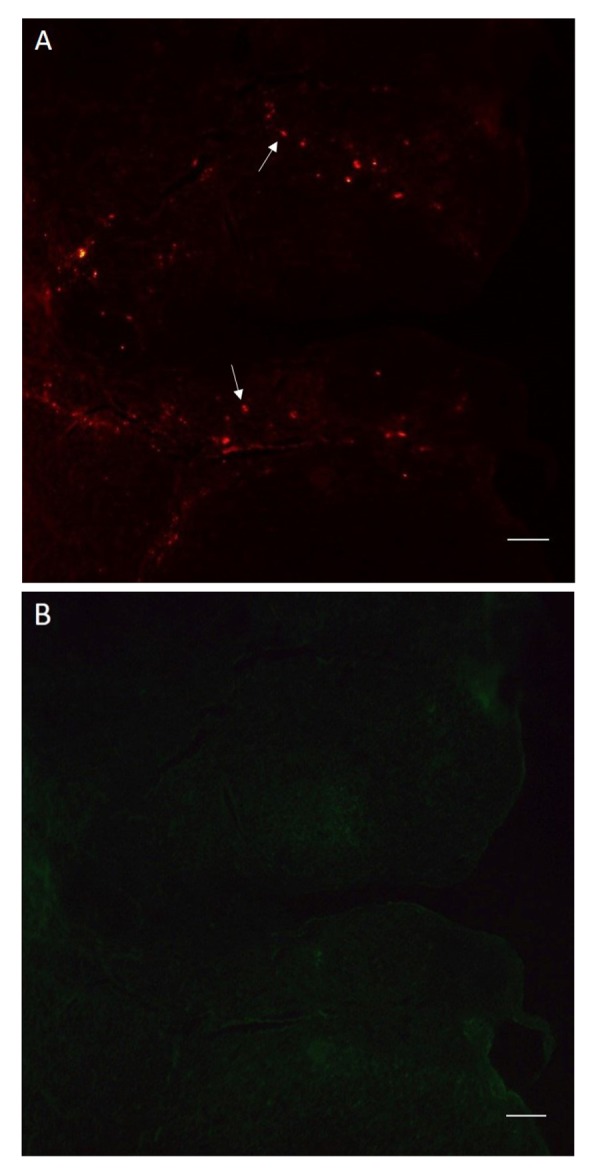
DiI labeled bone marrow stromal cells (BMSCs) in a section of ovary. **A.** The labeled BMSCs are visible as red spots and **B.** In the same section, the labeled BMSCs are not visible with green fluorescence (scale bars: 100 .m). Arrows show the labeled cells.

The transplanted BMSCs were labeled with dii, as red spots in the sections of ovaries (Fig .2). The results confirmed presence of the transplanted cells in the ovaries four weeks after transplantation. 

### Levels of serum estradiol and follicle-stimulating hormone

**Fig 3:**
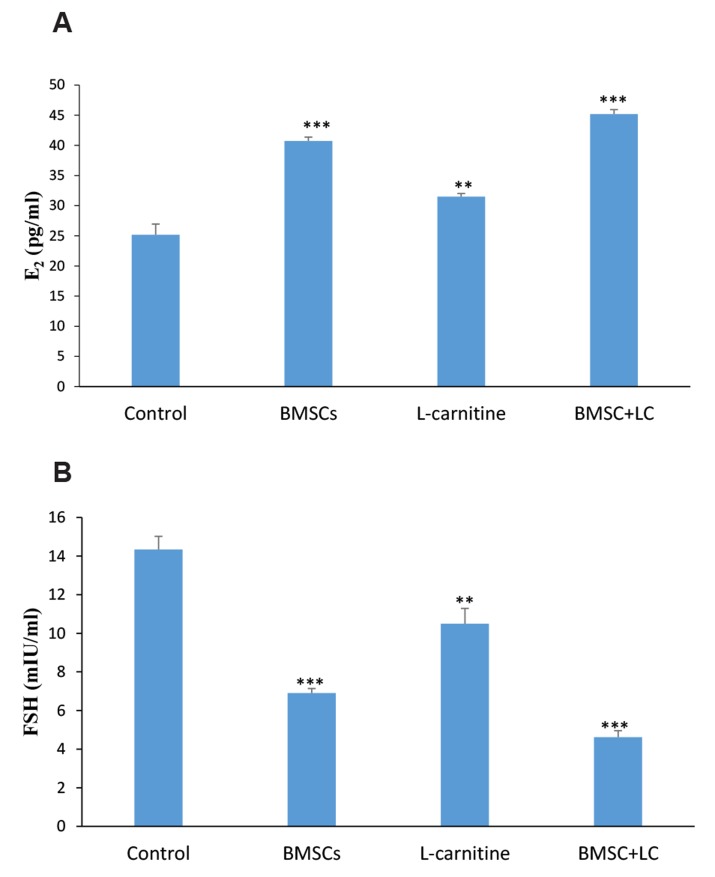
The levels of serum estradiol (E2) and follicle-stimulating hormone (FSH) in the experimental groups four weeks after treatment. **A.** The results of serum E2 level and** B.** The results of serum FSH level. **; P<0.01, ***; P<0.001 versus control group, and BMSC; Bone marrow stromal cells.

Hormonal examination was performed, by determining levels of serum E2 and FSH, four weeks after treatment. The results showed that levels of serum E2 in the BMSC+Lc co-administrated group (P<0.001), BMSC group (P<0.001) and Lc group (P<0.01) were significantly higher than the control group. The results of BMSC+Lc group were significantly higher than BMSC group (P<0.05) and Lc group (P<0.001). The results of BMSC group were significantly higher than Lc group (P<0.001, Table 1, Fig .3A). 

The levels of serum FSH in the BMSC+Lc co-administrated group (P<0.001), BMSC group (P<0.001) and Lc group (P<0.01) were significantly lower than the control group. The results of BMSC+Lc group were significantly lower than BMSC group (P<0.05) and Lc group (P<0.001). The results of BMSC group were significantly lower than Lc group (P<0.01, Table 1, Fig .3B).

### Histological evaluation of the ovaries

**Fig 4:**
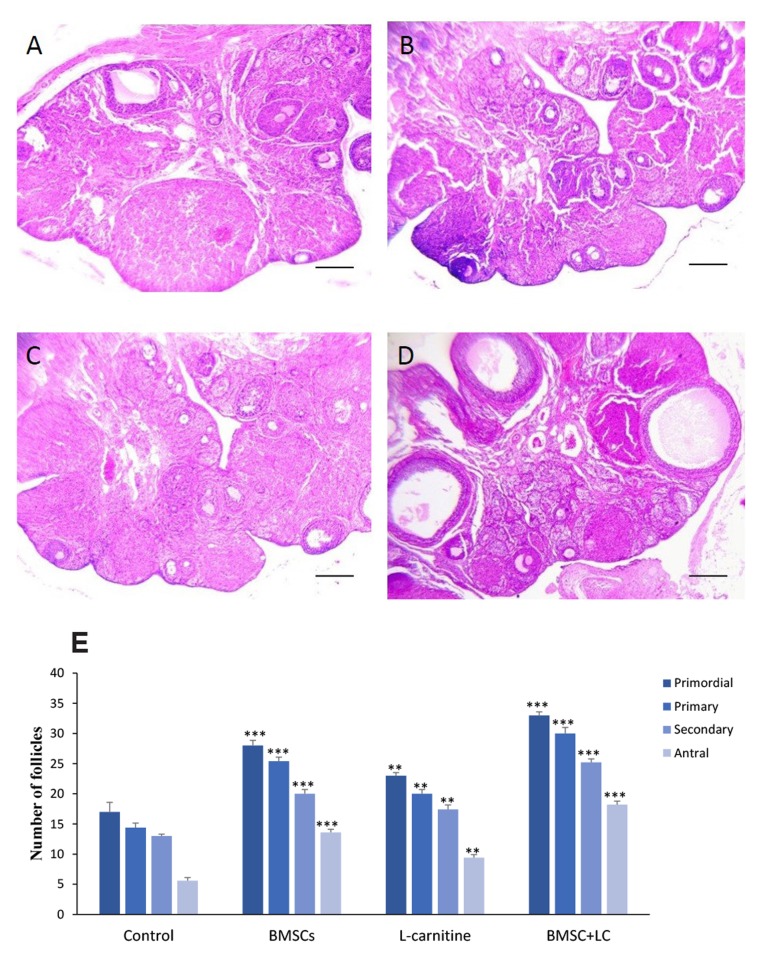
The number of follicles four weeks after treatment. H&E staining of ovaries in **A.** Control, **B.** BMSCs, **C.** L-carnitine, **D.** Co-administration of BMSC+Lc groups, and E. The number of follicles at different stages (scale bars: 200 .m). **; P<0.01, ***; P<0.001 versus control group, and BMSC; Bone marrow stromal cells.

H&E staining demonstrated that the number of all follicles in different stages was significantly higher in BMSC+Lc group compared to BMSC (P<0.01), Lc (P<0.001) and control groups (P<0.001). Findings showed that the number of all follicles in BMSC group was significantly more than Lc group (P<0.05, Table 1, Fig .4).

**Table 1:** Results of the hormonal, histological and expression of ovarian Bcl-2 and Bax proteins four weeks after treatment


Groups	Control	BMSCs	L-carnitine	BMSC+L-carnitine

E2 (pg/ml)	25.18 ± 1.769	40.74 ± 0.63^*^^*^^*^	31.48 ± 0.533^*^^*^	45.2 ± 0.728^*^^*^^*^
FSH (mIU/ml)	14.34 ± 0.682	6.9 ± 0.24^*^^*^^*^	10.5 ± 0.791^*^^*^	4.62 ± 0.338^*^^*^^*^
The number of ovarian follicles in different stages				
Primordial	17 ± 1.581	28.2 ± 0.86^*^^*^^*^	23.6 ± 0.51^*^^*^	33.2 ± 0.583^*^^*^^*^
Primary	14.4 ± 0.748	25.4 ± 0.678^*^^*^^*^	20 ± 0.707^*^^*^	30 ± 1.03^*^^*^^*^
Secondary	13 ± 0.316	20 ± 0.707^*^^*^^*^	17.4 ± 0.748^*^^*^	25.2 ± 0.583^*^^*^^*^
Antral	5.6 ± 0.51	13.6 ± 0.51^*^^*^^*^	9.4 ± 0.61^*^^*^	18.2 ± 0.583^*^^*^^*^
Expression of ovarian Bcl-2 protein	1.0 ± 0.0	1.473 ± 0.017^*^^*^^*^	1.89 ± 0.054^*^	2.347 ±0.167^*^^*^^*^
Expression of ovarian Bax protein	1.0 ± 0.0	0.806 ± 0.011^*^^*^^*^	0.392 ± 0.051^*^^*^	0.179 ± 0.027^*^^*^^*^
Bcl-2/Bax ratio	0.723 ± 0.047	1.12 ± 0.026^*^	1.143 ± 0.046^*^	4.018 ± 0.127^*^^*^^*^


Data are presented as mean ± SE. E2; Estradiol, FSH; Follicle-stimulating hormone, *; P<0.05, **; P<0.01, and ***; P<0.001 versus control group.

### Analysis of Bcl-2 and Bax in the ovaries

**Fig 5:**
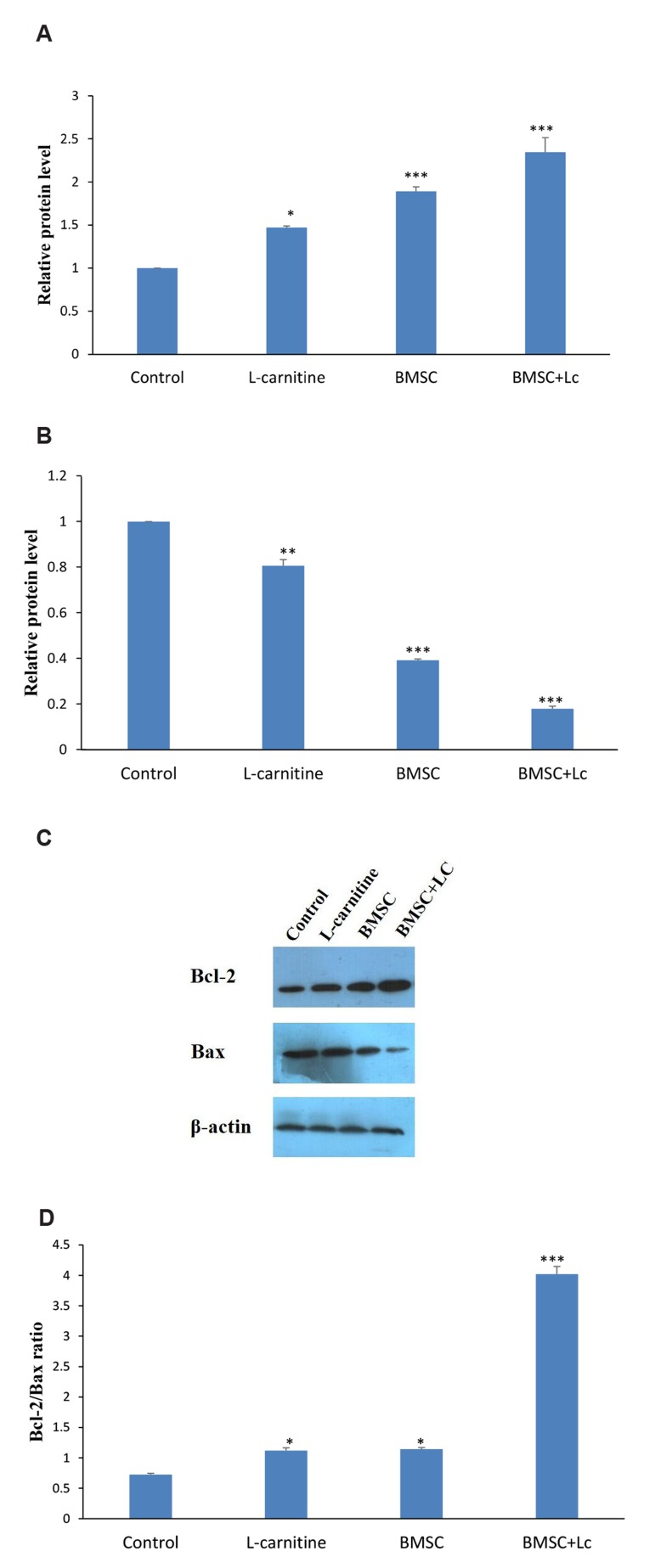
Analysis of Bcl-2 and Bax protein expressions by western blot assay four weeks after treatment. **A.** The expression of ovarian Bcl-2 protein, **B.** The expression of ovarian Bax protein, **C.** Immunoblot of Bcl-2, Bax and .-Actin proteins, and **D.** Bcl-2/Bax ratio in all groups. *; P<0.05, **; P<0.01, ***; P<0.001 versus control group.

Expression of ovarian Bcl-2 and Bax proteins was determined by Western blot. The results showed that Bcl-2 expression in the co-administration of BMSC+Lc (P<0.001), BMSC (P<0.001) and Lc groups (P<0.05) were significantly higher than the control group; while it was significantly higher than BMSC (P<0.05) and Lc groups (P<0.01) in BMSC+Lc. In addition, it was significantly higher in the BMSC, compared to Lc group (P<0.05). Bax expression in the BMSC+Lc co-administered group (P<0.001), BMSC group (P<0.001) and Lc group (P<0.001) were significantly lower than the control. It was significantly lower in the BMSC+Lc compared to BMSC (P<0.01) and Lc groups (P<0.001). Additionally, it was significantly lower than Lc group, in the BMSC group (P<0.05). The Bcl-2/Bax ratio was significantly increased in BMSC+Lc co-administered group, in comparison with the control group (P<0.001), BMSC group (P<0.001) and Lc group (P<0.001, Table 1, Fig .5).

## Discussion

Chemotherapy may damage the ovaries of girls and women, however, there are some ways to prevent from happening this. In this study, for the first time, we evaluated the effect of co-administration of BMSC+Lc on damaged ovaries after creating a chemotherapy model with cyclophosphamide in rat. Overall, the results showed that levels of serum E2 and FSH, number of follicles in different stages and expression of Bcl-2 and Bax proteins in BMSC+Lc co-administrated group were significantly more favorable than the control, BMSC and Lc groups.

Some studies have shown that BMSC and Lc may individually improve damaged ovaries (7, 8, 11). However, the effect of BMSC+Lc co-administration has never been applied for the same purpose. Comparing the effect of BMSC+Lc co-administration with either of them alone may introduce a novel clinical approach to the recovery of damaged ovaries by chemotherapy.

BMSCs, as a mesenchymal stem cell type, are a suitable candidate for cell therapy in damaged ovaries. Liu et al. (24) have reported that mesenchymal stem cells improve tissue repair chiefly via differentiation and paracrine effects. Several studies have shown that BMSCs produce some growth factors preventing cell apoptosis and repair the ovaries. Some of these growth factors include vascular endothelial growth factor (VEGF), insulin-like growth factor 1 (IGF-1), hepatocyte growth factor (HGF) and basic fibroblast growth factor (bFGF) (7, 8). VEGF is an angiogenic factor promoting formation of new capillary networks which provides nutrition for GCS (7, 8, 25). IGF-1 stimulates GC proliferation by regulating DNA replication of granulosa and theca cells. IGF-1 increases the function of gonadotropin hormones. Moreover, IGF-1 regulates aromatase activity, promotes follicular antrum formation and suppresses apoptosis in ovaries (7, 8). HGF promotes follicular maturation and inhibits apoptosis in ovarian follicles and GCS (7). Finally, bFGF works as a starter of folliculogenesis by inducing primordial follicle development (25). In this regard, Badawy et al. (26) showed that BMSCs could repair mouse ovarian insufficiency following cyclophosphamide induction, and Fu et al. (27) showed that overexpression of miR-21 in mesenchymal stem cells improved ovarian structure and function in rats with chemotherapy-induced ovarian damage. The results of our study are in agreement with these reports.

On the other hand, Lc as an antioxidant may also improve damaged ovaries. Zhang et al. (11) showed that Lc inhibits follicle apoptosis and increases the function of frozen-thawed ovaries in mice. However, the effect of Lc has not been assessed on rat ovaries damaged by a chemotherapy agent, cyclophosphamide. Some studies have shown that Lc has protective effects on other organs. For example, Aktoz et al. (28) showed that Lc has protective effects against testicular toxicity in rat, Mescka et al. (29) showed that Lc prevents oxidative stress in the brain of rats and Tousson et al. (30) showed that Lc has protective effects on rat cardiac injury. 

Lc plays an important role in fatty acid transport and lipid catabolism of mitochondria. Lc produces ATP by increasing .-oxidation of fatty acid. Hence, it can provide energy for follicular growth. Lc may also suppress apoptosis by increasing .-oxidation of fatty acids and reduce fatty acid toxicity. Moreover, accumulation of reactive oxygen species (ROS) in follicles leads to evacuation of the ATP reservoir, which decreases follicle quality. Lc, as a ROS scavenger and an energy generation facilitator, can be responsible for useful effects on follicular survival and ovarian function (11, 12, 31). In relation to this issue, Giorgi et al. (32) showed that Lc prevents miotic oocyte damage induced by follicular fluid from infertile women with mild endometriosis and Xu et al. (33) showed that Lc, during in vitro maturation of buffalo oocytes, improves oocyte quality. The results of our study are in agreement with these reports.

In addition, several studies have shown that Lc has favorable effects on mesenchymal stem cells. Fujisawa et al. showed that Lc suppresses apoptosis in BMSCs, due to restoration of mitochondrial activity and suppression of senescence induction by blocking TGF-., suggesting that Lc is involved in mitochondrial activation even in senescent cells (14). Lu et al. (15) showed that carnitine could affect differentiation rate of adult stem cells by regulating mitochondrial metabolism, and it may enhance tissue development. Farahzadi et al. (16) showed that Lc improves the lifespan of aged adipose tissue-derived human mesenchymal stem cells by overexpressing telomerase and lengthening telomeres.

Considering the beneficial effects of Lc on mesenchymal stem cells, in the present study, the combined effects of Lc and BMSCs were evaluated on the recovery of ovaries damaged by chemotherapy agent. We cultured BMSCs and transplanted them into the rat ovaries after creating the chemotherapy model. BMSCs expressed CD29, CD44 and CD90, but not CD34 and CD45. That was in agreement with other study (7). We labeled BMSCs with dii and transplanted them into the ovaries. It was shown that transplanted BMSCs could be present in the ovaries after four weeks. These results are in agreement with other report (21). To evaluate the ovarian function, levels of serum E2 and FSH were assessed by ELISA kit. To evaluate the ovarian structure, number of follicles at different stages was counted by HandE staining. Moreover, to evaluate apoptosis in the ovaries, expression of Bcl-2 and Bax proteins was measured by western blot, since the protein products of Bcl-2 and Bax genes are respectively described as anti-apoptotic and pro-apoptotic factors (23). Findings obtained from these evaluations showed that the results of BMSC and Lc groups were significantly more favorable than the control group. These results are in agreement with the other studies (8, 11, 23).

Indeed, the results of hormonal, histological and expression of Bcl-2 and Bax proteins were in the same direction and confirmed each other. So that, these results in BMSC+Lc co-administrated group were significantly more favorable than BMSC, Lc and control groups. The reasons are probably due to the combination of useful properties of BMSCs and Lc with different mechanisms of action in the restoration of ovaries after chemotherapy. In addition, considering that Lc has favorable effects on differentiation, increasing lifespan and decreasing apoptosis in BMSCs, it may increase survival of the transplanted BMSCs in the ovaries.

The results of BMSC group were significantly more favorable than Lc group. In the present study, considering that BMSCs were injected into the ovaries, these cells might produce some growth factors or might replace damaged cells in the ovaries (7-9). In this regard, Liu et al. (24) compared local and systemic administration of mesenchymal stem cells and reported that local administration of stem cells is the most efficient route for cell homing and immediate generation. So, probably due to these reasons, the recovery of damaged ovaries after chemotherapy with in situ transplantation of BMSCs were more favorable than intraperitoneal injection of Lc.

This study has some limitations which should be considered. The number of samples was small, so a larger sample size is required. Additionally, more research is necessary to clarify the molecular mechanisms underlying the function of BMSC and Lc to repair damaged ovary after chemotherapy. 

## Conclusion

The results of this study suggest that the effect of BMSC+Lc co-administration is probably more effective than the effect of their administrations individually on the recovery of ovaries damaged by cyclophosphamide chemotherapy agent in rat.
